# Real-Time PCR-Based Detection of Hepatitis E Virus in Groundwater: Primer Performance and Method Validation

**DOI:** 10.3390/ijms26157377

**Published:** 2025-07-30

**Authors:** Jin-Ho Kim, Siwon Lee, Eung-Roh Park

**Affiliations:** 1 Biomedical Research Institute, Dankook University Hospital, Cheonan 31116, Republic of Korea; cladonia@dankook.ac.kr; 2 Department of Chemistry, College of Science and Engineering, Dankook University, Cheonan 31116, Republic of Korea; 3 R&D Team, LSLK Co., Ltd., Gimpo 10111, Republic of Korea; siwonlee99@nate.com; 4 Water Use and Management Research Division, Water Environmental Research Department, National Institute of Environmental Research, Incheon 22689, Republic of Korea

**Keywords:** groundwater monitoring, hepatitis E virus (HEV), molecular diagnostics, real-time (rt) PCR, viral detection

## Abstract

Hepatitis E virus (HEV) is a leading cause of acute viral hepatitis and is primarily transmitted via contaminated water and food. Groundwater may also serve as a potential vector for HEV transmission. This study aimed to optimize real-time polymerase chain reaction (rtPCR) for the detection of HEV, employing both TaqMan probe- and SYBR Green-based methods. A total of 12 primer sets for the TaqMan probe-based method and 41 primer sets for the SYBR Green-based method were evaluated in order to identify the optimal combinations. Primer sets #4 (TaqMan probe-based) and #21 (SYBR Green-based) demonstrated the highest sensitivity and specificity, successfully detecting HEV in artificially spiked samples at 1 fg/μL. The TaqMan probe-based method facilitated rapid detection with minimized non-specific amplification, whereas the SYBR Green-based method allowed for broader primer exploration by leveraging melting curve analysis. Despite the absence of HEV detection in 123 groundwater samples, this study underscores the value of real-time PCR for environmental monitoring and paves the way for enhanced diagnostic tools for public health applications.

## 1. Introduction

Hepatitis E virus (HEV) is a non-enveloped RNA virus belonging to the *Hepeviridae* family that causes hepatitis, with four major genotypes (GT1, GT2, GT3, and GT4) [1,2]. Globally, HEV is predominantly distributed in areas with inadequate sewage management and poor hygiene and sanitation, with a particularly high prevalence in low-resource and developing countries [3,4]. The virus can be contracted primarily through ingestion of contaminated water or food and contact with infected animals [5,6,7]. Groundwater has been identified as a potential vector for infection [8]. While hepatitis E vaccines are currently available in some countries, they are not universally accessible worldwide, which has significant implications for public health [9,10]. Detecting HEV in environmental samples, particularly groundwater, is more challenging due to the low concentration of the target viruses and the presence of numerous interfering substances [11,12]. Consequently, numerous researchers have devised a range of biological molecular techniques for diagnosing HEV.

So far, testing has been conducted using a number of different methods, including reverse-transcription (RT) polymerase chain reaction (PCR), nested PCR, loop-mediated isothermal amplification (LAMP), droplet digital PCR (ddPCR), and clustered regularly interspaced short palindromic repeat (CRISPR)-based techniques [13,14,15,16,17]. These methods are constrained by their complexity, potential for contamination, non-specific reactions, and the need for specialized equipment [18,19,20]. However, real-time PCR (rtPCR) is a well-established technique that can be validated in real time, has a rapid detection time, and exhibits high sensitivity and specificity [21]. Moreover, the technique allows for not only qualitative but also quantitative analyses [22]. Accordingly, various primers and rtPCR techniques have been developed; however, the efficacy of each primer for rtPCR-based methods has not been assessed using groundwater samples.

Therefore, this study aimed to compare and evaluate the reaction efficiency of previously developed primers and probes for the diagnosis of HEV in groundwater samples, with a view to identifying the optimal combination.

## 2. Results

Following the amplification of 12 primer sets for the TaqMan probe-based method, primer sets #8 and #11 were found to be non-reactive, while primer sets 1 and 10 exhibited Cq values exceeding 35. Consequently, these four primer sets were initially excluded. Subsequently, the specificity of the primer sets was assessed using 22 reference viruses, and all primer sets demonstrated high specificity. The sensitivity test using a 10-fold serially diluted HEV plasmid revealed that primer set #4 could amplify the plasmid even at the lowest concentration (100 fg/μL) (Table 1).

A total of 41 combined primer sets were tested using the SYBR Green-based method to identify the optimal primer set. In this manner, negative samples exhibiting even minimal reactivity were excluded. Subsequently, ten primer sets were selected in accordance with the order of the lowest Cq value. The ten selected primer sets (# 4, 5, 6, 7, 13, 15, 20, 21, 22, and 23) were subsequently employed in sensitivity and specificity tests. The results demonstrated that the primer sets #6, #15, and #21 were capable of detecting positive samples at the lowest concentration (100 fg/μL) with Cq values ≤35 (Table 2). The three selected primer sets were employed in the specificity tests with 22 RefVs. The results demonstrated that each primer set exhibited a Cq value ≥35 (Supplementary Table S1). As a final confirmation step, a melting curve analysis was conducted to verify cross-reactivities. The results demonstrated a distinction between the melting peak of the HEV positive control (PC) and that of the RefVs’ amplification products (#6, PC 79.0 °C/RefV 80.5–84.0 °C). The melting curves for the HEV positive control and RefVs’ amplification products for the #6 primer set were as follows: #6, PC 79.0 °C/RefV 80.5 °C; #15, PC 78.5 °C/RefV 79.5 °C; #21, PC 78.0 °C/<75.0 °C and >80.5 °C (Supplementary Figure S1).

**Figure 1:**
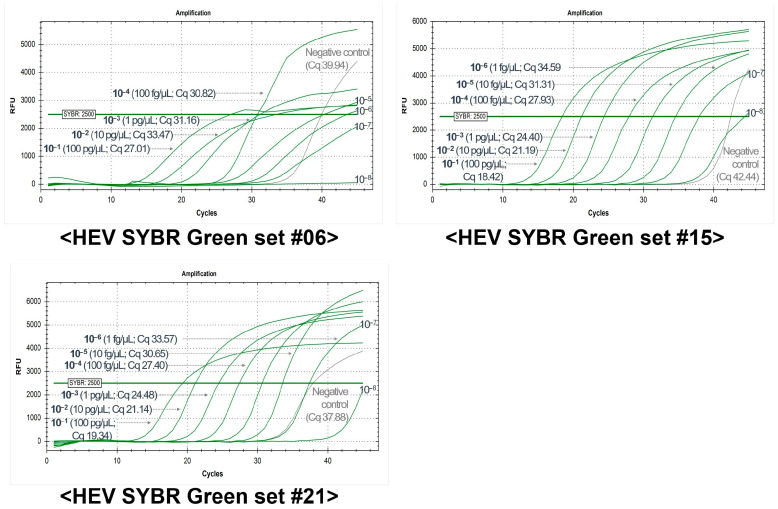
Sensitivity results of three HEV SYBR Green-based method primer sets using artificially spiked samples (ASSs).

A total of 123 groundwater samples were analyzed using three primer sets selected from the TaqMan probe- and the SYBR Green-based methods as the primary testing techniques. Although HEV was not detected in these environmental samples, to confirm the sensitivity of the method with environmental samples, an experiment was performed using artificially spiked samples (ASSs). The TaqMan probe-based primer set #4 was capable of detecting ASSs at a concentration of 1 fg/μL, with a reaction time of 78 min (Table 1). Additionally, three of the SYBR Green-based primer sets—#6, #15, and #21—were also able to detect ASSs at a concentration of 1 fg/μL in a manner comparable to the TaqMan probe-based primer set (Figure 1). The selected primer sets demonstrated high sensitivity and showed potential applicability for environmental monitoring.

**Figure 2:**
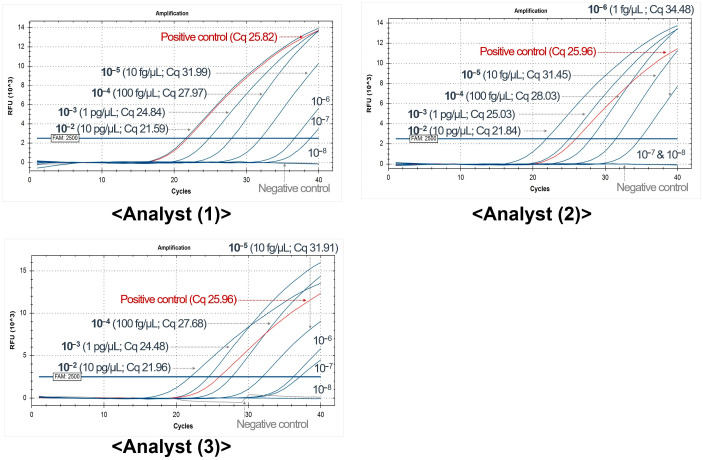
Validation results of the HEV TaqMan probe-based real-time (rt) PCR assay by three analysts.

To validate the experiment, three analysts repeated the sensitivity experiment using ASSs. The TaqMan probe-based method exhibited 10 times reduced sensitivity, with two of the three analysts detecting concentrations down to 10 fg/μL. However, one experimenter yielded identical results, detecting concentrations down to 1 fg/μL (Figure 2). To compare the two candidate primer sets (#15 and #21) using the SYBR Green-based method, three analysts performed two replicates and compared the Cq values, including the results provided by the developer. The results were averaged, with the maximum and minimum values for each concentration excluded. The results demonstrated that primer set #21 exhibited slightly greater sensitivity (Table 3). Consequently, the #21 primer set was selected for further analysis.

## 3. Discussion

This study aimed to compare and evaluate the detection efficiency of rtPCR-based tests for HEV in groundwater in Korea, utilizing previously reported primers and probe sets. The results demonstrated that both the TaqMan probe- and the SYBR Green-based methods have high sensitivity and specificity. The primer specificity was experimentally validated through in-silico testing using the BLAST with reference sequences (hepatitis E virus [taxid: 291484]). The test results confirmed that the selected primers specifically matched, displaying an identity of 100% with the reference sequences, thereby supporting the accuracy and reliability of our assay. Notably, primer set #4, using the TaqMan probe-based method, and primer set #21, using the SYBR Green-based method, demonstrated sensitivity for detection at a level as low as 1 fg/μL in ASSs. Furthermore, in contrast to previous studies on the detection of HEV, we assessed a multitude of primer combinations (41 sets for SYBR Green-based rtPCR) in addition to the previously documented primer sets, with the objective of exploring a broader range of possibilities. This contributed to the evaluation of the sensitivity and specificity of HEV detection, especially in complex matrices such as environmental samples. Particularly, utilizing this method for groundwater samples demonstrated its efficacy in assessing environmental samples. We also attempted to ascertain the relative advantages and weaknesses of the two rtPCR-based methods. The TaqMan probe-based method demonstrated a reduced probability of non-specific amplification and had a faster reaction time compared to the SYBR Green-based method, thereby promising greater efficiency in the analysis of environmental samples. Conversely, the SYBR Green-based method necessitated a supplementary melting curve analysis but afforded versatility to assess a broader spectrum of primer combinations.

A limitation of this study is that HEV was not detected in 123 actual groundwater samples, which may be indicative of either extremely low viral concentrations in those groundwater samples or degradation of the viral RNA due to environmental factors [23,24]. To address this, we successfully demonstrated the method sensitivity using groundwater samples artificially spiked with an HEV plasmid, underscoring the assay’s potential applicability to real-world environmental samples. Although artificially spiked samples are widely used to evaluate viral detection methods, they are known to have certain limitations, particularly regarding the overestimation of analytical sensitivity [25]. It is demonstrated that the recovery of viruses from environmental water samples can vary significantly depending on the characteristics of the sample. The recovery rates observed in this study ranged from 0.05% to 100%, and there was considerable variability across the trials. These findings suggest that preanalytical steps such as filtration and concentration may result in a substantial loss of viral particles, potentially leading to false-negative results even when viruses are present at low levels. Despite these limitations, artificially spiked samples remain a valuable tool for method standardization and performance evaluation under controlled conditions [26]. This is especially relevant in cases where naturally contaminated samples are unavailable or when the target virus, such as norovirus or hepatitis E virus, cannot be easily cultured. Rodríguez-Lázaro and colleagues underscored the technical complexity of detecting infectious virus particles, indicating that this task is not always feasible [26]. Consequently, they proposed the utilization of alternative infectivity assessment strategies. The application of artificial infection tests, encompassing surrogate viruses and animal models, provides critical insights into the feasibility of a method and the potential health risks associated with it. These tests serve as a crucial link between molecular detection and actual infectivity.

While HEV was not detected in the groundwater samples examined in this study, existing epidemiological evidence supports the ongoing circulation of HEV in South Korea. A nationwide seroprevalence study reported anti-HEV IgG positivity of 5.9% in the general population, with higher prevalence observed among individuals residing in rural areas or working in agriculture and fisheries, suggesting possible zoonotic transmission routes [27]. This is supported by the detection of anti-HEV antibodies in 18% of Korean blood donors and the identification of swine HEV genotype 3 in pig sera [28]. Furthermore, fecal shedding of HEV genotype 3 has been observed in approximately 17.5% of pigs from swine farms in South Korea [29]. These findings indicate the circulation of HEV in non-human reservoirs and foodborne matrices, suggesting the necessity of exercising caution when interpreting negative groundwater results. A multi-matrix surveillance approach, incorporating human serology, livestock monitoring, and food and water testing, is essential to comprehensively assess the public health risk posed by HEV in South Korea.

Traditional diagnostic methods, including conventional PCR, nested PCR, and LAMP, often suffer from potential contamination, non-specific amplification, complex procedures, and extended reaction times [30,31]. rtPCR provides significant improvements, including rapid detection, real-time validation, high specificity, and sensitivity. However, it requires specialized equipment and higher reagent costs, presenting potential limitations for its widespread use in resource-limited settings.

Nevertheless, this study contributes to the understanding of the environmental pathways of HEV infection and facilitates the improvement of molecular diagnostic tools for HEV monitoring, particularly in key resources such as groundwater.

## 4. Materials and Methods

The TaqMan probe- and SYBR green-based methods were both evaluated for rtPCR. The primer sets for both methods were collated by consulting the literature registered in PubMed from 2004 to 2023. In the event of multiple primer sets being identified, the most representative literature was selected based on the first report of primer information for inclusion (Table 4 and Table 5) [32,33,34,35,36,37,38,39,40,41,42,43,44,45,46,47,48,49]. For the primer sets employed in the SYBR Green-based method, a total of 19 forward and 11 reverse primers were combined from each literature source. These primer sets included those used in the TaqMan probe-based method. The collected primer sets were aligned against various HEV genotypes (GT1 to GT4) using the Basic Local Alignment Search Tool (BLAST 1.4.0) provided by NCBI. The primer alignment coverage indicated high conservation across different HEV genotypes. Furthermore, 41 combinations of primer sets with PCR amplicon sizes ranging from 70 to 150 nt were tested to provide greater diversity than that observed in previously reported primer sets (see Supplementary Table S2). The Cq analysis employed a threshold of 2500 relative fluorescence units (RFUs). A Cq value of less than 35 was taken to indicate a positive reaction. The conditions of the TaqMan probe-based rtPCR method were established following reference to the conditions described in the respective literature. The conditions for SYBR Green-based rtPCR were chosen following the manufacturer’s instructions (Bio-Rad, Hercules, CA, USA). For the TaqMan probe-based method, any primer set candidates that exhibited a non-specific reaction were excluded. Similarly, several primer sets in the SYBR Green-based method were excluded following confirmation by melting curve analysis. All PCR reactions were conducted using a CFX connect™ rtPCR system (Bio-Rad, Hercules, CA, USA). The TaqMan probe-based method was conducted using 10 μL of 2X Accupower^®^ Plus Dualstar™ qPCR master mix (Bioneer, Daejeon, Republic of Korea), 1 μL of forward primer (25 pmol/μL), 1 μL of reverse primer (25 pmol/μL) and probe (25 pmol/μL), 1 μL of template, and 5.6 μL of HiGene™ RNase-Free Water (BIOFACT Co., Ltd., Daejeon, Republic of Korea). The SYBR Green-based method was prepared using 10 μL of 2X iTaq Universal SYBR^®^ Green Supermix (Bio-Rad, Hercules, CA, USA), 1 μL of forward primer (25 pmol/μL), 1 μL of reverse primer (25 pmol/μL), 1 μL of template, and 7 μL of HiGene™ RNase-Free Water (BIOFACT Co., Ltd., Daejeon, Republic of Korea).

A synthesized HEV plasmid was used as a positive control to evaluate the rtPCR methods. The HEV positive control was prepared by Macrogen Co., Ltd. (Seoul, Republic of Korea) using the ORF2 gene sequence (accession number [FJ763142.1], 1168 nucleotides [nt]) from the National Centre for Biotechnology Information (NCBI). To determine the analytical sensitivity, a 1 ng/μL solution of the HEV plasmid was diluted 10-fold, from 10^−1^ (100 pg/μL) to 10^−8^ (10 ag/μL), and used as the template for the subsequent reaction. For testing the analytical specificity of the method, 22 reference viruses (RefVs) were used. The names, genotypes, groups, inserted genes, and concentrations of these viruses are shown in Table 3. When both rtPCR methods were conducted using groundwater samples, 3 μL of 1X SL™ non-specific reaction inhibitor (LSLK Co., Ltd., Incheon, Republic of Korea) was added. In all PCR reactions, HiGene™ RNase-Free Water (BIOFACT Co., Ltd., Daejeon, Republic of Korea) was used as a negative control. Additionally, 1 pg/μL of HEV plasmid was used as a positive control.

A total of 123 groundwater samples were collected from 47 administrative regions across 11 metropolitan cities and provinces in South Korea. The sampling wells were selected annually based on multiple criteria, including installation year, capacity, usage, and baseline microbiological indicators, such as E. coli and nitrate nitrogen. The samples were collected biannually (in the first and second halves of each year) across multiple provinces to ensure broad temporal and spatial coverage without geographical or seasonal bias. These samples were collected based on the national norovirus groundwater monitoring network operated by the National Institute of Environmental Research (NIER) under the Ministry of Environment from 2021 to 2024. The sampling procedures were carried out in accordance with the established protocols outlined in NIER Notice No. 2017-50, which serves as the standard for virus sampling methodologies [50]. At each site, a team of at least three trained personnel conducted environmental assessments, disinfected the wells following sufficient flushing, and measured water quality parameters including temperature, pH, turbidity, and residual chlorine. Pretreatment steps, including turbidity removal and dechlorination, were implemented when deemed necessary. The filtration process, which was conducted over a period of 3–5 h, involved the use of a controlled-flow housing unit equipped with a virus adsorption filter, resulting in the filtration of approximately 500 L of groundwater. The filters were transported to the laboratory on ice, and virus elution and concentration determination were performed within the recommended holding time according to the standardized recovery protocol defined by NIER Notice No. 2017-50 [51]. In addition, the virus concentration method used in this study is also based on a standardized protocol established by NIER Notice No. 2017-50 [50]. This method was developed by benchmarking the U.S. EPA Method 1615 and aligning it with ISO 15216-1:2017. It has been validated for its recovery efficiency and reproducibility. Although a process control virus was not added to individual samples, the procedure was conducted within the framework of the national groundwater virus surveillance system, which includes multiple layers of quality assurance. These include mandatory training and on-site inspections for field staff, implementation of an analytical quality control program, and regular interlaboratory proficiency testing. The absence of internal process controls is offset by the validation and verification practices embedded in this standardized method.

The groundwater samples were eluted and concentrated, and total RNA was extracted from the final concentrate in accordance with the instructions provided in the RNeasy Mini Kit manual (Qiagen, Hilden, Germany). The RNA samples of the reference viruses, including the extracted total RNA, were subjected to cDNA synthesis using ReverTra Ace-α-^®^ (Toyobo, Osaka, Japan). The nucleic acids of the RefVs were sourced from the National Institute of Environmental Science, Ministry of Environment. Similarly, the synthetic gene sequences of the RefVs in plasmid form were obtained from NCBI and synthesized for use as the HEV positive control by Macrogen Co., Ltd. (Seoul, Republic of Korea) (Table 6). If HEV was not amplified in the environmental samples, the prepared HEV positive control (1 ng/μL) was artificially infected with a mixed solution of 10 randomly selected groundwater samples and diluted 10-fold in order to evaluate the analytical sensitivity of the two real-time PCR methods with environmental samples. The artificial infection step was performed after the sample concentration step. The above-mentioned validation experiments were conducted by three researchers.

## 5. Conclusions

In conclusion, the selected primer set could be further applied to assess the public health significance of HEV in both clinical and environmental surveillance and could be used for the initial monitoring of HEV infection in the future.

## Figures and Tables

**Table 1:** Comparative analysis of the specificity, sensitivity, and running time of HEV TaqMan probe-based real-time (rt) PCR assays *.

Number of HEV-TP	Specificity		Plasmid-Based Sensitivity (2500 RFU) ^b^								Artificial Spiking-Based Sensitivity (2500 RFU) ^c^								Running Time (min) ^d^
	Target	22 Refs. ^a^	100 pg	10 pg	1 pg	100 fg	10 fg	1 fg	100 ag	10 ag	100 pg	10 pg	1 pg	100 fg	10 fg	1 fg	100 ag	10 ag	
1	35.0 <	N/A	37.10	39.37	N/A	N/A	N/A	N/A	N/A	N/A	34.94	37.31	N/A	N/A	N/A	N/A	N/A	N/A	110.0
2	**O**		33.84	30.73	36.56	40.14	41.12	43.89	N/A	N/A	29.20	30.91	32.36	34.84	35.61	38.54	43.34	42.93	85.0
3	**O**		26.93	29.86	30.75	40.31	N/A	N/A	N/A	N/A	N/A	28.76	29.78	31.44	34.59	38.38	44.13	N/A	100.0
4 **^e^**	**O**		23.45	23.94	28.15	32.82	35.18	38.58	N/A	N/A	23.69	25.34	24.73	28.64	30.26	33.61	36.61	38.03	78.0
5	**O**		16.32	28.22	32.76	36.45	38.97	43.36	N/A	N/A	23.66	20.15	25.70	31.10	33.96	36.67	40.00	43.57	114.0
6	**O**		26.61	26.75	31.68	38.26	38.64	42.43	42.06	N/A	30.42	27.11	32.35	33.87	33.38	36.68	40.47	N/A	86.0
7	**O**		25.48	28.26	33.53	39.42	N/A	N/A	N/A	N/A	24.27	26.83	28.36	30.96	33.94	37.99	40.91	N/A	95.0
8	N/A		N/A	N/A	N/A	N/A	N/A	N/A	N/A	N/A	N/A	N/A	N/A	N/A	N/A	N/A	N/A	N/A	100.0
9	**O**		26.48	30.64	35.47	41.14	42.48	N/A	N/A	N/A	29.47	27.81	29.19	32.78	35.18	38.83	43.16	N/A	100.0
10	35.0<		36.71	N/A	N/A	N/A	N/A	N/A	N/A	N/A	N/A	N/A	N/A	N/A	N/A	N/A	N/A	N/A	140.0
11	N/A		N/A	N/A	N/A	N/A	N/A	N/A	N/A	N/A	N/A	N/A	N/A	N/A	N/A	N/A	N/A	N/A	76.0
12	**O**		32.15	34.42	N/A	N/A	N/A	N/A	N/A	N/A	30.00	35.69	37.18	37.17	N/A	N/A	N/A	N/A	76.0

* All results are expressed as mean Cq values derived from two replicates.; RFU, relative fluorescence unit; **O**, specific reaction; N/A, not applicable; bold, Cq values < 35.0. ^a^ Refs., nucleic acid of reference viruses; Refs-#1, enteric *adenovirus* (eAdV) 40 (eAdV-40) DNA (1 pg/μL); Refs-#2, eAdV-41 DNA (100 copies); Refs-#3, *Aichivirus* A1 (AiV-A1) plasmid [NC_001918, VP3-VP1 (2740–3918), 2AB (4400–5199) and 3CD (6038–8011)] (each, 1 pg/μL); Refs-#4, *Astrovirus* (AstV) plasmid [JN887820, ORF 1b-2 (3800–6685)] (1 pg/μL); Refs-#5, *Coxsackievirus* (CoxV) A6 (CoxV-A6) cDNA (100 copies); Refs-#6, CoxV-A24 cDNA (1 pg/μL); Refs-#7, CoxV-B1 cDNA (100 copies); Refs-#8, CoxV-B5 cDNA (100 copies); Refs-#9, *Echovirus* (EchoV) 5 (EcoV-5) cDNA (100 copies); Refs-#10, EcoV-11 cDNA (1 pg/μL); Refs-#11, EcoV-22 cDNA (1 pg/μL); Refs-#12, *Enterovirus* (EV) 68 (EV-68) cDNA (100 copies); Refs-#13, EV-71 cDNA (100 copies); Refs-#14, *Hepatitisvirus* A (HAV) cDNA (1 pg/μL); Refs-#15, *Norovirus* (NoV) GI (NoV-GI) plasmid [JQ388274, ORF1/VP1 (5283–5673)] (1 pg/μL); Refs-#16, NoV-GII cDNA (100 copies); Refs-#17, *Orthoreovirus* (OrV) plasmid [NC_013231.1, segment S (146–975)] (1 pg/μL); Refs-#18, *Parechovirus* (PeV-A) plasmid [NC_001897.1, 5′UTR/VP3-VP1-2A (411–613, 2078–3110)] (1 pg/μL); Refs-#19, *Poliovirus* type 3 (PV-type3) plasmid [AY184221.1, 5′UTR/VP2/VP1/3C (159–646, 1403–1611, 2503–2567, 3252–3376, 5559–5644)] (1 pg/μL); Refs-#20, *Reovirus* (ReV) cDNA (1 pg/μL); Refs-#21, *Rotavirus* (RV-A) cDNA (100 copies); Refs-#22, *Sapovirus* GI (SaV-GI) plasmid [KP298674.1, NS7-VP1 (4440–6439)] (1 pg/μL). ^b^ Cq value; 10-fold serially diluted template from 1 ng/μL. ^c^ The artificial spiking-based sensitivity test was conducted by randomly selecting 10 HEV-negative groundwater samples, combining them, and utilizing this mixture as a diluent for a 10-fold dilution of the HEV template (1 ng/μL). ^d^ The reaction times were based on the use of the CFX Connect Real-Time PCR (Bio-Rad, USA). ^e^ Final selected primer sets.

**Table 2:** Comparison of the sensitivities of primer sets using the SYBR Green-based method for the HEV real-time (rt) PCR assay.

HEV-SYBR Set #	Plasmid-Based Sensitivity (2500 RFU, Cq Value)								Running Time with Melting Curve Analysis (min)
	100 pg	10 pg	1 pg	100 fg	10 fg	1 fg	100 ag	10 ag	
4	26.51	25.33	31.54	36.09	37.30	N/A	N/A	N/A	Less than 110
5	26.37	28.49	34.85	38.16	40.33	42.17	40.58	41.54	
6 *	21.61	24.20	29.50	33.81	36.04	39.58	N/A	N/A	
7	24.61	24.23	30.82	36.15	36.40	39.44	43.06	N/A	
13	24.60	24.13	30.26	35.18	36.25	N/A	N/A	38.69	
15 *	25.53	24.56	30.53	35.00	39.59	44.15	N/A	N/A	
20	27.05	25.78	31.79	36.28	38.75	40.90	N/A	N/A	
21 *	25.00	24.77	30.50	34.87	35.77	41.77	37.53	36.95	
22	N/A	N/A	N/A	N/A	N/A	N/A	N/A	N/A	
23	27.34	25.56	31.02	36.92	36.06	38.54	41.60	N/A	

* Candidate PCR primer set.; RFU, relative fluorescence unit; N/A, not applicable; bold, Cq values < 35.0; under var, Cq = 35.0.

**Table 3:** Validation results of HEV SYBR Green-based primer sets #15 and #21 by three analysts.

HEV-SYBR_Set #	Division		Artificial Spiking-Based Sensitivity (2500 RFU, Cq Value)								
			100 pg	10 pg	1 pg	100 fg	10 fg	1 fg	100 ag	10 ag	Neg
#15	Developer		18.42	21.19	24.40	27.93	31.31	34.59	37.92	44.43	42.44
	Repeat (1)	Analyst 1	21.00	21.55	24.66	28.04	30.70	34.65	38.04	40.43	42.72
		Analyst 2	25.55	24.87	26.37	28.97	31.36	34.63	38.93	39.87	N/A
		Analyst 3	22.42	25.19	28.23	29.79	31.88	34.89	39.53	41.83	N/A
	Repeat (2)	Analyst 1	21.76	22.35	25.42	28.85	32.68	35.78	40.60	43.76	N/A
		Analyst 2	22.87	22.69	25.59	29.00	32.15	36.12	40.39	44.16	N/A
		Analyst 3	22.46	22.08	25.46	28.54	32.32	36.29	39.90	38.91	38.87
	Minimum		18.42	21.19	24.40	27.93	30.70	34.59	37.92	38.91	38.87
	Maximum		25.55	25.19	28.23	29.79	32.68	36.29	40.60	44.43	44.47
	Average (7 Cq values)		22.07	22.85	25.73	28.73	31.77	35.28	39.33	41.91	(N/A)
	Average (5 Cq values) *		21.37	22.38	25.23	28.52	31.59	35.08	39.08	41.41	(N/A)
#21	Developer		19.34	21.14	24.48	27.40	30.65	33.57	37.39	N/A	37.88
	Repeat (1)	Analyst 1	20.94	21.15	24.75	27.81	30.50	34.24	37.55	38.09	N/A
		Analyst 2	22.46	22.48	25.38	28.11	30.81	35.03	37.53	40.21	N/A
		Analyst 3	23.03	25.22	27.19	29.16	31.52	34.37	37.31	38.71	44.47
	Repeat (2)	Analyst 1	22.06	23.48	26.66	28.99	32.59	35.66	38.49	37.04	N/A
		Analyst 2	25.07	23.54	26.04	28.57	32.45	36.99	N/A	39.53	N/A
		Analyst 3	35.69	25.76	26.96	28.97	32.37	35.91	39.98	40.42	41.96
	Minimum		19.34	21.14	24.48	27.40	30.50	33.57	37.31	37.04	37.88
	Maximum		35.69	25.76	27.19	29.16	32.59	36.99	39.98	40.42	44.47
	Average (7 Cq values)		24.08	23.25	25.92	28.43	31.56	35.11	38.04	39.00	N/A
	Average (5 Cq values) *		21.76	22.75	25.67	28.28	31.35	34.73	37.56	38.65	N/A

* The maximum and minimum values for each concentration were excluded.; RFU, relative fluorescence unit; N/A, not applicable; bold, Cq values < 35.0.

**Table 4:** Information on TaqMan probe-based real-time (rt) PCR method for detecting hepatitis E virus.

#	Primer (TaqMan Probe)				Location		Product Size (nt)	PCR Conditions ^b^					Reference ^c^
	Type	Name	Sequence (5′→3′)	Length (bp)	Start ^a^	End ^a^		1st DN	DN	AN	EX	Cycle	
1	F	Sense primer	GACAGAATTRATTTCGTCGGCTGG	24	6336	6359	73	95 °C, 10 min	95 °C, 15 s	60 °C, 60 s		50	[32,33]
	R	HEV_R	CCYTTRTCYTGCTGNGCRTTCTC	23	6386	6408							
	Probe	Probe	FAM-GTYGTCTCRGCCAATGGCGAGC-BHQ1	22	6385	6406							
2	F	JVHEV-F	GGTGGTTTCTGGGGTGAC	18	5299	5316	70	95 °C, 15 min	95 °C, 10 s	55 °C, 20 s	72 °C, 15 s	45	[34]
	R	JVHEV-R	AGGGGTTGGTTGGATGAA	18	5351	5368							
	Probe	JVHEV-P	FAM-TGATTCTCAGCCCTTCGC-BHQ1	18	5322	5339							
3	F	HevMrsFwd	AATTRATTTCGTCGGCYGG	19	6341	6359	73	95 °C, 10 min	95 °C, 15 s	60 °C, 60 s		45	[35]
	R	HevMrsRev	ACWGTCGGCTCGCCATTG	18	6396	6413							
	Probe	HevMrsFam	FAM-ACTCYCGCCCSGTYGTCTCA-BHQ1	20	6374	6393							
4	F	Forward	CGGTGGTTTCTGGGGTGA	18	5298	5315	75	95 °C, 3 min ^d^	95 °C, 10 s	55 °C, 40 s		40	[36]
	R	Reverse	GCGAAGGGGTTGGTTGGA	18	5355	5372							
	Probe	Probe	FAM-TGATTCTCAGCCCTTCGC-BHQ1	18	5322	5339							
5	F	forward primer	CGGCGGTGGTTTCTGG	16	5295	5310	75	95 °C, 15 min	94 °C, 15 s	60 °C, 60 s		50	[37]
	R	reverse primer	AAGGGGTTGGTTGGATGAATA	21	5349	5369							
	Probe	probe	FAM-TGACAGGGTTGATTCTCAGCCCTTCG-BHQ1	26	5313	5338							
6	F	HEV_F	CGACAGAATTGATTTCGTCGGC	22	6335	6356	121	95 °C, 15 min	95 °C, 15 s	60 °C, 35 s		45	[33]
	R	HEV_R	CCYTTRTCYTGCTGNGCRTTCTC	23	6433	6455							
	Probe	HEV_TM	FAM-TYGGCTCGCCATTGGCYGAGAC-BHQ1	22	6386	6407							
7	F	HEV-F	CCGACAGAATTRATTTCGTCGGC	23	6344	6366	115	95 °C, 2 min	95 °C, 15 s	60 °C, 60 s		45	[38]
	R	HEV-R	ATACCCTTRTCYTGCTGIGCRTTCTC	26	6433	6458							
	Probe	HEV-P	FAM-GTCTCAGCCAATGGCGAG-BHQ1	18	6388	6405							
8	F	Forward	GGTGGTTTCTGGGGTGAC	18	5299	5316	73	95 °C, 10 min	95 °C, 15 s	60 °C, 60 s		45	[39]
	R	Reverse	CGAAGGGGTTGGTTGGATG	19	5353	5371							
	Probe	Probe	FAM-ATTCTCAGCCCTTCGCAATCCCCT-BHQ1	24	5324	5347							
9	F	HEV25	CGGTGGTTTCTGGGGTGA	18	5298	5315	75	95 °C, 10 min	95 °C, 15 s	60 °C, 60 s		45	[40]
	R	HEV26	GCRAAGGGRTTGGTTGG	17	5356	5372							
	Probe	HEV-MGB	FAM-ATTCTCAGCCCTTCGC-BHQ1	16	5324	5339							
10	F	HEV-5260-F	CGGTGGTTTCTGGGGTGAC	19	5298	5316	71	95 °C, 5 min	95 °C, 15 s	60 °C, 60 s	65 °C, 60 s	45	[41]
	R	HEV-5330-R	AGGGGTTGGTTGGATGAATATAG	23	5346	5368							
	Probe	HEV-5280-T	FAM-GGGTTGATTCTCAGCCCTTCGC-BHQ1	22	5318	5339							
11	F	HEV-5260-F	CGGTGGTTTCTGGGGTGAC	19	5298	5316	71	95 °C, 5 min	95 °C, 15 s	60 °C, 35 s		45	[42,43]
	R	HEV-5330-R	AGGGGTTGGTTGGATGAATATAG	23	5346	5368							
	Probe	E-Probe(M)	FAM-GGTTGATTCTCAGCCCTTCGC-BHQ1	21	5319	5339							
12	F	HEV-5260-F	GGTGGTTTCTGGGGTGAC	18	5299	5315	70	95 °C, 5 min	95 °C, 15 s	55 °C, 35 s		45	[42,43]
	R	JVHEV-R(M)	AAGGGGTTGGTTGGATGAATA	21	5349	5368							
	Probe	JVHEV-P(M)	FAM-TTGATTCTCAGCCCTTCGC-BHQ1	19	5321	5348							

^a^ Location was calculated based on FJ763142.1 (NCBI accession number); ^b^ 1st DN, first denaturation; DN, denaturation; AN, annealing; EX, extension; ^c^ KMFDS, Korea Ministry of Food and Drug Safety; MSS, Ministry of SMEs and Startups (Korea); ^d^ five cycles (95 °C for 10 s, 50 °C for 20 s, and 72 °C for 30 s) were conducted before starting the main reaction.

**Table 5:** Primer information on SYBR Green-based real-time (rt) PCR method for detecting hepatitis E virus.

#	Type	Name	Sequence (5′→3′)	Length (nt)	Location *		#
					Start	End	
1	Forward	HEV-05_08Matsubayashi_F	CGGCGGTGGTTTCTGG	16	5295	1	[37]
2	Forward	HEV-13_04Orrù_F	GCGGTGGTTTCTGGGG	16	5297	2	[44]
3	Forward	HE361	GCRGTGGTTTCTGGGGTGAC	20	5297	3	[45]
4	Forward	HEV-5260-F	CGGTGGTTTCTGGGGTGAC	19	5298	4	[45]
5	Forward	HEV-02_06Jothikumar_F	GGTGGTTTCTGGGGTGAC	18	5299	5	[34]
6	Forward	JVHEV-F(M)	GGTGGTTTCTGGGGTGA	17	5299	6	[46]
7	Forward	HE366	GYTGATTCTCAGCCCTTCGC	20	5320	7	[46]
8	Forward	HEV_SLNL-F	TCCCCYATATTCATCCAACCAA	22	5342	8	[47]
9	Forward	HEV-F1	TCCCCTATATTCATCCAACCAA	22	5342	9	[48]
10	Forward	HEV_SLNL-NF	GAYCARKCCCAGCGCCCCG	19	5449	10	[47]
11	Forward	HEV-F2	GAYCARKCCCAGCGCCCC	18	5449	11	[48]
12	Forward	F-3156N	AATTATGCCCAGTACCGGGTTG	22	5725	12	[39]
13	Forward	HE044	CAAGGHTGGCGYTCKGTTGAGAC	23	5950	13	[45]
14	Forward	HE110-2	GYTCKGTTGAGACCWCBGGBGT	22	5960	14	[49]
15	Forward	F-3158N	GTTATGCTTTGCATACATGGCT	22	6010	15	[49]
16	Forward	ORF2 BOVF2	CYGTYGTSTCRGCCAATGG	19	6321	16	[47]
17	Forward	ORF2 BOVF1	GGBCTNCCGACAGAATTRAT	20	6328	17	[47]
18	Forward	HEV-06_09Adlhoch_F	CGACAGAATTGATTTCGTCGGC	22	6335	18	[33]
19	Forward	HEV-03_07Colson_F	AATTRATTTCGTCGGCYGG	19	6341	19	[35]
20	Reverse	HE041	TTMACWGTCRGCTCGCCATTGGC	23	6394	20	[13]
21	Reverse	HEV-03_07Colson_R	ACWGTCGGCTCGCCATTG	18	6396	21	[35]
22	Reverse	HEV-09_12Pas_R	GCRAAGGGRTTGGTTGG	17	6393	22	[40]
23	Reverse	HEV-R_NL	RGCCGACGAAATYAATTCTGTC	23	6335	23	[47]
24	Reverse	HEV-R (=R-3159N)	RGCCGACGAAATYAATTCTGTC	22	6336	24	[47]
25	Reverse	HE364	CTGGGMYTGGTCDCGCCAAG	20	5441	25	[45]
26	Reverse	HE363	GMYTGGTCDCGCCAAGHGGA	20	5437	26	[45]
27	Reverse	HEV-08_12KMFDS_R	CGAAGGGGTTGGTTGGATG	19	5353	27	[39]
28	Reverse	HEV-02_06Jothikumar_R	AGGGGTTGGTTGGATGAA	18	5351	28	[34]
29	Reverse	HEV-5330-R	AGGGGTTGGTTGGATGAATATAG	23	5346	29	[42]
30	Reverse	JVHEV-R(M)	AGGGGTTGGTTGGATGAATA	20	5349	30	[42]

* Location was calculated based on FJ763142.1 (NCBI accession number).

**Table 6:** Information on hepatitis E virus and 22 reference viruses as positive control or for testing the analytical specificity.

#	Division and #	Virus	Genotype	Acronym	Group *	Gene	NCBI Accession # *	Length (nt) *	Conc.
1	Target	*Hepatitisvirus*	E	HEV	Plasmid	ORF2	FJ763142.1	1168	1 ng/μL based dilutes
2	Refs. #01	Enteric *Adenovirus*	40	eAdV-40	DNA	-	-	-	1 pg/μL
3	Refs. #02	Enteric *Adenovirus*	41	eAdV-41	DNA	-	-	-	1 pg/μL
4	Refs. #03	*Aichivirus*	A1	AiV-A1	Plasmid	VP3-VP1/2AB/3CD	NC_001918.1	3953	1 pg/μL
5	Refs. #04	*Astrovirus*	-	AstV	Plasmid	ORF1b-2	JN887820.1	2886	1 pg/μL
6	Refs. #05	*Coxsackievirus*	A6	CoxV-A6	cDNA	-	-	-	100 copies
7	Refs. #06	*Coxsackievirus*	A24	CoxV-A24	cDNA	-	-	-	1 pg/μL
8	Refs. #07	*Coxsackievirus*	B1	CoxV-B1	cDNA	-	-	-	100 copies
9	Refs. #08	*Coxsackievirus*	B5	CoxV-B5	cDNA	-	-	-	100 copies
10	Refs. #09	*Echovirus*	5	EcoV-5	cDNA	-	-	-	100 copies
11	Refs. #10	*Echovirus*	11	EcoV-11	cDNA	-	-	-	1 pg/μL
12	Refs. #11	*Echovirus*	22	EcoV-22	cDNA	-	-	-	1 pg/μL
13	Refs. #12	*Enterovirus*	68	EV-68	cDNA	-	-	-	100 copies
14	Refs. #13	*Enterovirus*	71	EV-71	cDNA	-	-	-	100 copies
15	Refs. #14	*Hepatitisvirus*	A	HAV	cDNA	-	-	-	1 pg/μL
16	Refs. #15	*Norovirus*	GI	NoV-GI	Plasmid	ORF1/VP1	JQ388274.1	391	1 pg/μL
17	Refs. #16	*Norovirus*	GII	NoV-GII	cDNA	-	-	-	100 copies
18	Refs. #17	*Orthoreovirus*	-	OrV	Plasmid	Segment S	NC_013231.1	830	1 pg/μL
19	Refs. #18	*Parechovirus*	A	PeV-A	Plasmid	5′UTR/VP3-VP1-2A	NC_001897.1	1237	1 pg/μL
20	Refs. #19	*Poliovirus*	Type3	PV-type3	Plasmid	5′UTR/VP2/VP1/3C	AY184221.1	983	1 pg/μL
21	Refs. #20	*Reovirus*	-	ReV	cDNA	-	-	-	1 pg/μL
22	Refs. #21	*Rotavirus*	A	RV-A	cDNA	-	-	-	100 copies
23	Refs. #22	*Sapovirus*	GI.1	SaV-GI	Plasmid	NS7-VP1	KP298674.1	2000	1 pg/μL

* Information on synthesized gene location: HEV (ORF2 gene, NCBI accession number FJ763142.1, 5293–6460), AiV-A1 [VP3-VP1 (NC_001918.1, 2740–3918)/2AB (4400–5199)/3CD (6038–8011)], AstV (ORF1b-2, JN887820.1, 3800–6685), NoV-GI (ORF1/VP1, JQ388274.1, 5283–5673), OrV (Segment S, NC_013231.1, 146–975), PeV-A [5′UTR (NC_001897.1, 411–613)/VP3-VP1-2A (2078–3110), PV-type3 [5′UTR (AY184221.1, 159–646)/VP2 (1403–1611)/VP1 (2503–2567 and 3252–3376)/3C (5559–5644)], SaV-GI (NS7-VP1, KP298674.1, 4440–6439).

## Data Availability

All data are included in the manuscript. The HEV and reference virus genomes were obtained from GenBank, and the GenBank accession number for each sequence is provided in the manuscript. The original contributions presented in the study are included in the article/Supplementary Materials. Further inquiries can be directed to the corresponding author/s.

## Supplementary Materials

The following supporting information can be downloaded at: https://www.mdpi.com/article/10.3390/ijms26157377/s1.
