# Supplementary material for: Real-Time PCR-Based Detection of Hepatitis E Virus in Groundwater: Primer Performance and Method Validation
Source: Int J Mol Sci. 2025 Jul 30;26(15):7377. doi: 10.3390/ijms26157377 (PMC12347669; doi:10.3390/ijms26157377)
Supplement: Supplementary file 1 [file ijms-26-07377-s001.zip › 03-2. Supplementary Tables S1 and S2_v2.pdf]

Supplementary Table S1. The specificity test of primer sets designed for the detection of hepatitis E virus (HEV) through a SYBR Green-based real-time PCR method

Template (conc.)	HEV SYBR Green set #		
	#06	#15	#21
eAdV-40, DNA (1 pg/μL)	40.45	N/A	41.52
eAdV-41, DNA (100 copies)	40.46	44.64	44.95
AiV-A1, Plasmid (1 pg/μL)	N/A	43.39	44.87
AstV, Plasmid (1 pg/μL)	44.32	N/A	40.64
CoxV-A6, cDNA (100 copies)	43.66	N/A	N/A
CoxV-A24, cDNA (1 pg/μL)	42.64	39.97**	43.08
CoxV-B1, cDNA (100 copies)	42.49	N/A	43.82
CoxV-B5, cDNA (100 copies)	N/A	N/A	43.03
EcoV-5, cDNA (100 copies)	N/A	N/A	43.16
EcoV-11, cDNA (1 pg/μL)	N/A	N/A	43.38
EcoV-22, cDNA (1 pg/μL)	40.23	N/A	43.51
EV-68, cDNA (100 copies)	N/A	44.73	43.45
EV-71, cDNA (100 copies)	38.47*	44.29	38.78*
HAV, cDNA (1 pg/μL)	41.07	N/A	40.68
NoV-GI, Plasmid (1 pg/μL)	N/A	N/A	N/A
NoV-GII, cDNA (100 copies)	40.71	44.02	N/A
OrV, Plasmid (1 pg/μL)	44.79	N/A	43.95
PeV-A, Plasmid (1 pg/μL)	41.79 (or N/A)**	N/A	42.66
PV-type3, Plasmid (1 pg/μL)	41.6	N/A	39.74*
ReV, cDNA (1 pg/μL)	40.12	N/A	41.9
RV-A, cDNA (100 copies)	42.94	N/A	42.24
SaV-GI, Plasmid (1 pg/μL)	42.04	N/A	39.88*

All data were analyzed using 2,500 RFU and the Cq criteria (<35.0). When the Cq result was over 35, all of these were negative by melting curve analysis. *Average of replicate experiments; **Average of triplicate experiments

Supplementary Table S2. Information on SYBR Green-based real-time PCR primer combinations and amplification product sizes for the detection of hepatitis E virus (HEV).

Combination (Length; bp)	HE041	HEV-03_07Colson_R	HEV-09_12Pas_R	HEV-R_NL	HEV-R (=R-3159N)	HE364	HE363	HEV-08_12KMFDS_R	HEV-02_06Jothikumar_R	HEV-5330-R	JVHEV-R(M)
**HEV-05_08Matsubayashi_F**	1,122	1,119	1,115	1,063	1,063	166	162	**77 (#1)***	**74 (#2)**	**74 (#3)**	**74 (#4)**
**HEV-13_04Orrù_F**	1,120	1,117	1,113	1,061	1,061	164	160	**75 (#5)**	**72 (#6)**	**72 (#7)**	**72 (#8)**
**HE361**	1,120	1,117	1,113	1,061	1,061	164	160	**75 (#9)**	**72 (#10)**	**72 (#11)**	**72 (#12)**
**HEV-5260-F**	1,119	1,116	1,112	1,060	1,060	163	159	**74 (#13)**	**71 (#14)**	**71 (#15)**	**71 (#16)**
**HEV-02_06Jothikumar_F**	1,118	1,115	1,111	1,059	1,059	162	158	**73 (#17)**	**70 (#18)**	**70 (#19)**	**70 (#20)**
**JVHEV-F(M)**	1,118	1,115	1,111	1,059	1,059	162	158	**73 (#21)**	**70 (#22)**	**70 (#23)**	**70 (#24)**
**HE366**	1,097	1,094	1,090	1,038	1,038	**141 (#25)**	**137 (#26)**	52	49	49	49
**HEV_SLNL-F**	1,075	1,072	1,068	1,016	1,016	**119 (#27)**	**115 (#28)**	30	27	27	27
**HEV-F1**	1,075	1,072	1,068	1,016	1,016	**119 (#29)**	**115 (#30)**	30	27	27	27
**HEV_SLNL-N**	968	965	961	909	909	12	8	-77	-80	-80	-80
**HEV-F2**	968	965	961	909	909	12	8	-77	-80	-80	-80
**F-3156N**	692	689	685	633	633	-264	-268	-353	-356	-356	-356
**HE044**	467	464	460	408	408	-489	-493	-578	-581	-581	-581
**HE110-2**	457	454	450	398	398	-499	-503	-588	-591	-591	-591
**F-3158N**	407	404	400	348	348	-549	-553	-638	-641	-641	-641
**ORF2 BOVF2**	**96 (#31)**	**93 (#32)**	**89 (#33)**	37	37	-860	-864	-949	-952	-952	-952
**ORF2 BOVF1**	**89 (#34)**	**86 (#35)**	**82 (#36)**	30	30	-867	-871	-956	-959	-959	-959
**HEV-06_09Adlhoch_F**	**82 (#37)**	**79 (#38)**	**75 (#39)**	23	23	-874	-878	-963	-966	-966	-966
**HEV-03_07Colson_F**	**76 (#40)**	**73 (#41)**	69	17	17	-880	-884	-969	-972	-972	-972

*The Number in the designation describes a combination of primer sets. Product size of selected primer set is within the range of 70 – 150 nt.
