# Supplementary figures and images for: Real-Time PCR-Based Detection of Hepatitis E Virus in Groundwater: Primer Performance and Method Validation
Source: Int J Mol Sci. 2025 Jul 30;26(15):7377. doi: 10.3390/ijms26157377 (PMC12347669; doi:10.3390/ijms26157377)

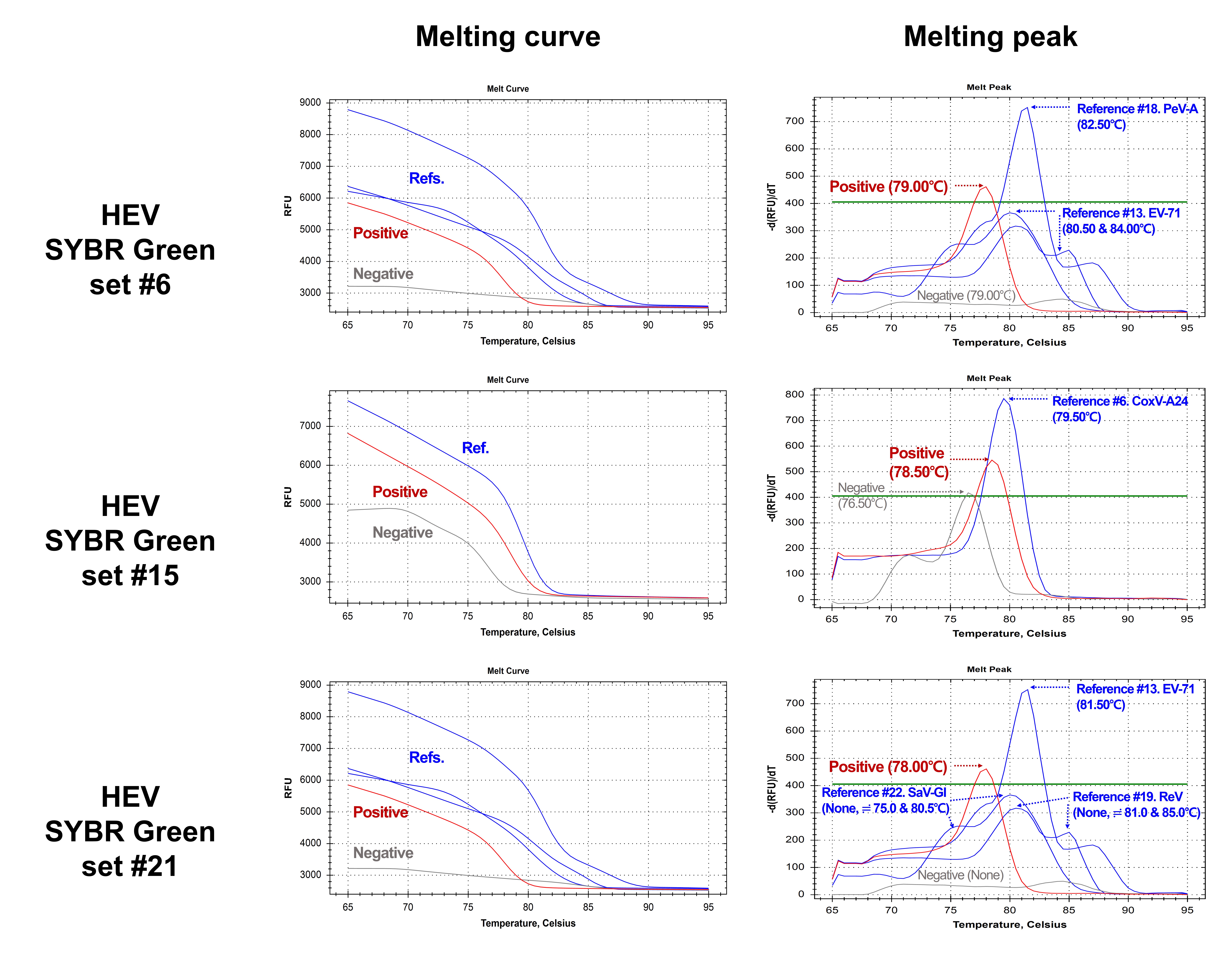

Supplement: Supplementary file 1 [file ijms-26-07377-s001.zip › 04. Supplementary Figure S1.jpg]
